# Systematic review: preoperative psychological factors and total hip arthroplasty outcomes

**DOI:** 10.1186/s13018-022-03355-3

**Published:** 2022-10-17

**Authors:** John P. O’Connor, Paige Holden, Joel J. Gagnier

**Affiliations:** 1 School of Public Health, University of Michigan, 1415 Washington Heights, Ann Arbor, MI 48109 USA; 2 University of Connecticut School of Medicine, Farmington, CT USA; 3 Departments of Orthopaedic Surgery and Epidemiology, University of Michigan, Ann Arbor, MI USA

**Keywords:** Total hip arthroplasty, Mental health, Depression, Anxiety, Systematic review, Operative outcomes

## Abstract

**Background:**

Total hip arthroplasties (THA) are cost-effective interventions for patients with osteoarthritis refractory to physical therapy or medical management. Most individuals report positive surgical outcomes with reduction in pain and improved joint function. Multiple recent studies demonstrated the influence of patient mental health on surgical success. We sought to determine the relationship between patient preoperative psychological factors and postoperative THA outcomes, specifically pain and function.

**Methods:**

PubMed, EMBASE and Cochrane Reviews databases were queried using terms “(mental OR psychological OR psychiatric) AND (function OR trait OR state OR predictor OR health) AND (outcome OR success OR recovery OR response) AND total joint arthroplasty).” A total of 21 of 1,286 studies fulfilled inclusion criteria and were included in the review. All studies were analyzed using GRADE and Risk of Bias criteria.

**Results:**

Overall, compared to cohorts with a normal psychological status, patients with higher objective measures of preoperative depression and anxiety reported increased postoperative pain, decreased functionality and greater complications following THA. Additionally, participants with lower self-efficacy or somatization were found to have worse functional outcomes.

**Conclusions:**

Preoperative depression, anxiety and somatization may negatively impact patient reported postoperative pain, functionality and complications following THA. Surgeons should consider preoperative psychological status when counseling patients regarding expected surgical outcomes.

**Level of evidence:**

3.

**Supplementary Information:**

The online version contains supplementary material available at 10.1186/s13018-022-03355-3.

## Introduction

Total hip arthroplasties (THA) effectively improve quality of life for individuals with end-stage osteoarthritis [1]. Most patients have positive surgical results including improved pain, strength and range of motion. Unfortunately, a subset of individuals undergoing THA report unsatisfactory outcomes not necessarily attributable to operative technique, presurgical pain levels or loss of function [2]. Since the late 1990s, attention has been paid to the role of social and psychological factors in contributing to these suboptimal outcomes [3].

Multiple studies have evaluated the effect of concomitant psychological factors on changes in patient reported outcome measures following THA [4–24]. Most commonly, anxiety and depression have been investigated in relation to postoperative pain and function [4, 5, 7–10, 13–16, 18–21, 23, 24]. Attitudinal factors such as self-efficacy, optimism/pessimism, resilience and surgical fear as well as personality traits including self-care and pain catastrophizing have also been studied. Unfortunately, the current literature lacks an updated systematic review evaluating the role of these characteristics on hip pain and function following THA. By reviewing published THA studies, we sought to fill this gap by investigating the impact of patient psychological status on THA outcomes. We hypothesize individuals with worse preoperative mental functional status will have poorer outcomes following THA compared to those with normal psychological function.

## Methods

The PRISMA (Preferred Reporting Items for Systematic Reviews and Meta-Analyses) guidelines were followed when preparing this manuscript (Additional file 1) [25].

### Search strategy

PubMed, EMBASE and the Cochrane Library were searched with the following terms through November 12, 2021: (mental OR psychological OR psychiatric) AND (function OR trait OR state OR predictor OR health) AND (outcome OR success OR recovery OR response) AND total joint arthroplasty. We also reviewed reference lists of relevant review articles and included papers.

### Inclusion and exclusion criteria

All randomized or observational cohort studies involving patients 18 years of age or older published in any language that investigated the role of psychological variables as predictors or effect modifiers of outcomes following THA were included. The outcomes of interest included hip pain, physical and psychological function, and complications/adverse events after THA.

Search results were independently reviewed by two individuals. Publication titles and abstracts were screened. Full text was reviewed if more information was needed to determine whether studies fulfilled all inclusion criteria. Reasons for study exclusion were documented. Reviewer disagreements were resolved by discussion or, when needed, by a third party. References of the included studies were also reviewed for additional sources not found via database searches.

**Fig. 1:**
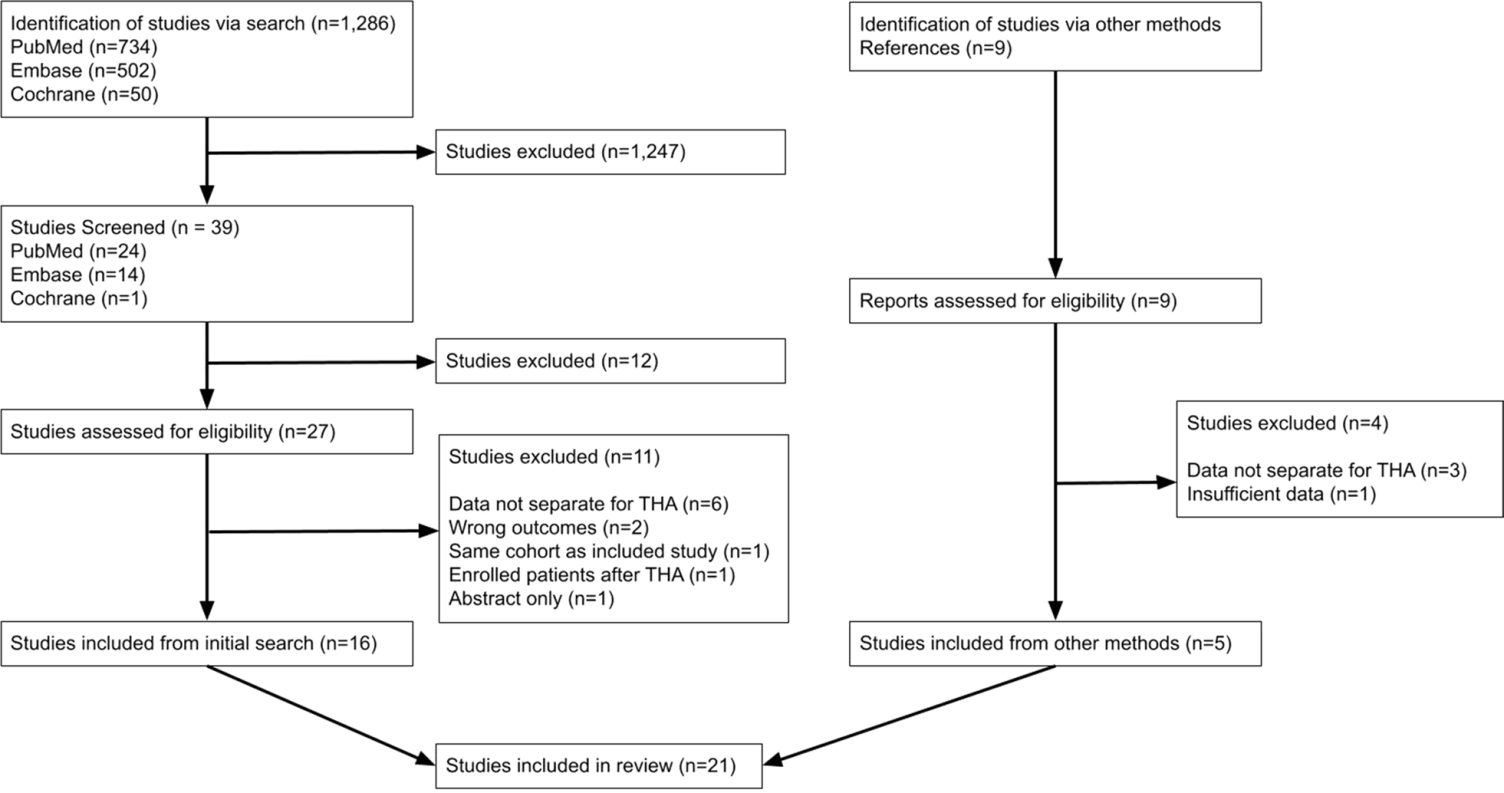
Flow diagram of study selection process

A total of 1,286 publications were screened with 21 meeting inclusion criteria (Additional file 2). The main reasons for study exclusion were lack of preoperative psychological assessments or desired postoperative outcome measures (Fig. 1). A total of 12,925 adult participants (55% female) were included across the 21 papers. All publications were nonrandomized cohort trials (15 prospective, 6 retrospective). A total of 13 studies were conducted in Europe, while the remaining eight were completed in North America.

### Assessment of study quality

The risk of bias of the included nonrandomized cohort studies was assessed by two reviewers using accepted criteria [26]. Each of the possible sources of bias was explicitly judged as being fulfilled (Y), not fulfilled (N) or unknown (?) due to incomplete information or inadequate reporting (Table 1).

**Table 1:** Risk of bias assessment for observational studies

	Was the outcome absent at the start of the study?	Are all members of the cohort equally capable of developing the outcome?	Was the outcome measurement performed in the same manner with similar intensity in the groups being compared?	Were the groups similar at baseline?	Did the authors perform analyses adjusting for known confounders?	Were all the withdrawals and dropsout described and accounted for?	Did the authors describe other possible sources of bias or confounders?
Badura-Brzoza [4]	Yes	Yes	Yes	Yes	No	No	No
Benditz [5]	Yes	Yes	Yes	?	No	Yes	Yes
Brembo [6]	Yes	Yes	Yes	?	Yes	Yes	Yes
Duivenvoorden [7]	Yes	Yes	Yes	Yes	Yes	Yes	Yes
Etcheson [8]	Yes	Yes	Yes	Yes	Yes	No	Yes
Galea [9]	Yes	Yes	Yes	No	Yes	Yes	Yes
Hassett [10]	Yes	Yes	Yes	Yes	Yes	No	Yes
Hossain [11]	Yes	Yes	Yes	No	Yes	No	Yes
Jaiswal [12]	Yes	Yes	Yes	No	Yes	Yes	No
Lindner [13]	Yes	Yes	Yes	?	No	Yes	Yes
Mercurio [14]	Yes	Yes	Yes	?	No	Yes	Yes
Negrini [15]	Yes	Yes	No	?	No	No	Yes
Pinto [16]	Yes	Yes	Yes	Yes	No	No	Yes
Quintana [17]	Yes	Yes	Yes	No	Yes	Yes	Yes
Rasouli [18]	Yes	Yes	Yes	Yes	Yes	No	Yes
Riediger [19]	Yes	Yes	Yes	No	No	Yes	No
Rolfson [20]	Yes	Yes	Yes	Yes	Yes	No	No
Salmon [21]	Yes	Yes	Yes	Yes	No	Yes	No
Singh [22]	Yes	Yes	Yes	Yes	Yes	Yes	Yes
Tarakji [23]	Yes	Yes	Yes	No	No	No	Yes
Trinh [24]	Yes	Yes	Yes	No	Yes	Yes	Yes

*Yes* this criterion was fulfilled, *No* this criterion was not fulfilled; ?, it was not possible to determine whether or not the criterion was fulfilled due to underreporting or unclear language

The risk of bias assessment scores were utilized to determine whether effect size differed by study quality. Studies were divided into two groups based on the number of criteria fulfilled [27]. The high risk of bias group (< 5 criteria fulfilled) included 4 studies, while 17 studies comprised the low risk of bias group (5–7 criteria fulfilled).

### Data collection and abstraction

#### Administration

All relevant papers were exported into Zotero where duplicates were removed and articles were reviewed for inclusion eligibility.

#### Data extraction

Two reviewers extracted data from the included studies. Publication information such as title, authors, year and country of origin were recorded. Study characteristics including design, inclusion/exclusion criteria, follow-up duration, outcome domains (e.g., pain, function, subsequent complications/adverse events), outcome measures (e.g., HHS, WOMAC, SF-36), description of outcome events and relationship between psychological determinant and outcome domains were tabulated. Patient data such as age, gender, psychological status, cohort composition and psychological variables measured (e.g., depression, anxiety, pain catastrophizing, etc.) were recorded.

### Synthesis methods

GRADE (Grading of Recommendations Assessment, Development and Evaluation) was used to gauge the overall evidence quality of the included studies [28]. We downgraded an initial rating of low quality by one level for serious problems regarding risk of bias, inconsistency, indirectness and imprecision [28]. The GRADE assessments were done separately for individual outcome domains and further by outcome measures and follow-up duration.

## Results

Due to the heterogeneity of patient populations, variables measured and outcomes used, we could not perform a meta-analysis and instead performed a systematic review, using rigorous and well accepted methods assessing the overall quality and levels of evidence.

### Preoperative psychological variables and postoperative pain

Overall, 12 studies reported the association of preoperative psychological variables with postoperative pain following THA [6–8, 10, 13, 14, 16, 17, 19, 20, 22, 23]. Most (9/12, 75%) authors confirmed preoperative depression, anxiety or other mental health disorders resulted in increased pain after surgery compared to patients without psychiatric illness. When stratified by follow-up duration, postoperative pain persisted in patients with depression, anxiety or other mental health disorders compared to control subjects (Table 2). No strong relationship was found for personality traits such as optimism or pessimism as well as pain catastrophizing and self-efficacy.

**Table 2:** Study conclusions

Risk of bias score	Author, year	n	Follow-up duration	Psychological variable(s)	Main conclusion(s)
*Pain study conclusions*					
< 5	Riediger [19]	79	2 months	Depression, somatization	↑ Depression/somatization associated with ↑ pain
	Tarakji [23]	44	3,12 months	Depression	↑ Depression associated with ↑ pain
5–7	Brembo [7]	223	3 months	Self-efficacy	↓ Self-efficacy associated with ↑ pain
	Duivenvoorden [7]	140	3,12 months	Depression/anxiety	↑ Anxiety/depression associated with ↑ pain
	Etcheson [8]	93	48 h	Depression	↑ Depression has no significant impact on pain
	Hassett [10]	862	3,6 months	Depression/anxiety	↑ Anxiety/depression associated with ↑ pain
	Lindner [13]	44	6,12 weeks	Depression/anxiety	↑ Anxiety associated with ↑ pain; ↑ Depression has no significant impact on pain
	Mercurio [14]	40	12 months	Depression/anxiety	↑ Anxiety/depression ↑ pain
	Pinto [16]	64	48 h	Depression/anxiety, optimism	↑ Anxiety/depression have no significant impact on pain; ↑ Optimism associated with ↓ pain
	Quintana [17]	788	6 months, 2 years	Mental health	↓ Mental health associated with ↑ pain
	Rolfson [20]	6,158	1 year	Depression/anxiety	↑ Anxiety/depression associated with ↑ pain
	Singh [22]	441	2 years	Pessimism	↑ Pessimism has no significant impact on pain
*Function study conclusions*					
< 5	Badura-Brzoza [4]	184	6 months	Depression/anxiety	↑ Anxiety/depression have no significant impact on function
	Negrini [15]	40	3,12 days	Depression/anxiety	↑ Anxiety/depression have no significant impact on function
	Riediger [19]	79	2 months	Depression, somatization	↑ Depression/somatization associated with ↓ function
	Tarakji [23]	44	3,12 months	Depression	↑ Depression associated with ↓ function
5–7	Benditz [5]	50	1,5 weeks	Depression/anxiety	↑ Anxiety/depression associated with ↓ function
	Brembo [6]	223	3 months	Self-efficacy	↓ Self-efficacy associated with ↓ function
	Duivenvoorden [7]	140	3,12 months	Depression/anxiety	↑ Anxiety/depression associated with ↓ function
	Galea [9]	627	3 months, 1,7 years	Depression/anxiety	↑ Anxiety/depression associated with ↓ function
	Hossain [11]	762	1,5 years	Distress	↑ Distress associated with ↓ function
	Lindner [13]	44	6,12 weeks	Depression/anxiety	↑ Anxiety associated with ↓ function; ↑ Depression has no significant impact on function
	Quintana [17]	788	6 months, 2 years	Mental health	↓ Mental health associated with ↓ function
	Salmon [21]	102	1,6 months	Depression/anxiety	↑ Anxiety associated with ↓ function
	Singh [22]	441	2 years	Pessimism	↑ Pessimism associated with ↓ function
	Trinh [24]	48	1 year	Depression	↑ Depression associated with ↓ function
*pain and function study conclusions*					
5–7	Jaiswal [12]	677	1 year	Mental health	↓ Mental health associated with ↓ function and ↑ pain
*other study conclusions*					
5–7	Rasouli [18]	964	NR	Complications	↑ Anxiety/depression associated with ↑ complications

*NR* Not recorded; ↑ increased; ↓ decreased

All high risk of bias (2/2) studies demonstrated a significant relationship between a psychological variable and increased pain. Most (8/10) of the studies deemed low risk of bias presented a significant relationship.

The most common study shortcomings were not adjusting for confounders and failing to address patient attrition. Retrospective studies were less likely to account for patient dropout [8, 11, 18, 20, 23].

### Preoperative psychological variables and postoperative function

Postoperative function was evaluated in 14 studies [4–7, 9, 11, 13, 15, 17, 19, 21–24] and decreased in THA recipients with abnormal psychological variables in 12/14 (86%). Depression, anxiety, distress, pessimism, somatization and low self-efficacy were associated with lower functional status after THA. Postoperative functional status remained low when evaluated in the short-, medium- and long-term follow-up periods (Table 2). No relationship was found for mood or personality traits.

Half of the high risk of bias studies (2/4) found a significant impact of psychological variables on function. All low risk of bias studies (10/10) presented a significant association between psychological variables and decreased function.

Rasouli et al. [18] assessed postoperative complications and reported depression and anxiety to be predictors of increased complications following THA, specifically anemia and infection (Table 2).

Jaiswal et al. [12] evaluated postoperative pain and function with a combined WOMAC measure and found mental health impairment to be associated with increased pain and decreased function (Table 2).

### GRADE

Evidence was assessed using the GRADE criteria for observational studies separately for each outcome measure. All groups began with a low quality of evidence. A total of 14 publications included a functional outcome (Table 3), 12 evaluated pain (Table 4), and one assessed both pain and function (Table 5).

**Table 3:** GRADE function

Certainty assessment							No. of patients
Follow-up	Study design	Risk of bias	Inconsistency	Indirectness	Imprecision	Quality	
*WOMAC PF*							
0–1 week	None						
1–12 weeks	4 observational studies	None	Not serious	Not serious	Not serious	Low	264
12 weeks–6 months	3 observational studies	None	Not serious	Not serious	Not serious	Low	907
6 months–2 years	1 observational study	None	Not serious	Not serious	Not serious	Low	310
Over 2 years	None						
*sf-36 pcs*							
0–1 week	None						
1–12 weeks	3 observational studies	None	Not serious	Not serious	Not serious	Low	1033
12 weeks–6 months	1 observational study	None	Not serious	Not serious	Not serious	Low	184
6 months–2 years	2 observational studies	None	Not serious	Not serious	Not serious	Low	844
Over 2 years	1 observational study	None	Not serious	Not serious	Not serious	Low	627
*hhs*							
0–1 week	1 observational study	None	Not serious	Not serious	Not serious	Low	50
1–12 weeks	2 observational studies	None	Not serious	Not serious	Not serious	Low	960
12 weeks–6 months	None						
6 months–2 years	1 observational study	None	Not serious	Not serious	Not serious	Low	800
Over 2 years	1 observational study	None	Not serious	Not serious	Not serious	Low	627
*HOOS*							
0–1 week	None						
1–12 weeks	None						
12 weeks–6 months	None						
6 months–2 years	1 observational study	None	Not serious	Not serious	Not serious	Low	140
Over 2 years	none						
*Gait speed*							
0–1 week	1 observational study	Serious	Not serious	Serious	Serious	Very low	40
1–12 weeks	1 observational study	Serious	Not serious	Serious	Serious	Very low	40
12 weeks–6 months	None						
6 months–2 years	None						
Over 2 years	None						
*OHS*							
0–1 week	None						
1–12 weeks	None						
12 weeks–6 months	None						
6 months–2 years	1 observational study	None	Not serious	Not serious	Not serious	Low	908
Over 2 years	1 observational study	None	Not serious	Not serious	Not serious	Low	762
*PROMIS-10*							
0–1 week	None						
1–12 weeks	None						
12 weeks–6 months	None						
6 months–2 years	1 observational study	None	Not serious	Serious	Not serious	Low	48
Over 2 years	None						
*Other*							
0–1 week	None						
1–12 weeks	None						
12 weeks–6 months	None						
6 months–2 years	1 observational study	None	Not serious	Serious	Not serious	Very losw	441
Over 2 years	None						

*WOMAC* Western Ontario and McMaster University Osteoarthritis Index, *Pain* pain subscale, *PF* Physical functioning subscale, *SF-36 PCS* Short-form health survey physical component score, *HHS* Harris hip score, *HOOS* Hip dysfunction and osteoarthritis outcome score, *OHS* Oxford hip score, *PROMIS-10* Patient reported outcomes measurement information system-10

**Table 4:** GRADE pain

Certainty assessment							No. of patients
Follow-up	Study design	Risk of bias	Inconsistency	Indirectness	Imprecision	Quality	
*WOMAC pain*							
0–1 week	None						
1–12 weeks	3 observational studies	None	Not serious	Not serious	Not serious	Low	167
12 weeks–6 months	2 observational studies	None	Not serious	Not serious	Not serious	Low	813
6 months–2 years	2 observational studies	None	Not serious	Serious	Not serious	Very low	350
Over 2 years	None						
*VAS*							
0–1 week	1 observational study	None	Not serious	Not serious	Not serious	Low	93
1–12 weeks	None						
12 weeks–6 months	None						
6 months–2 years	2 observational studies	None	Not serious	Not serious	Not serious	Low	6198
Over 2 years	None						
*SF-36 pain*							
0–1 week	None						
1–12 weeks	2 observational studies	None	Not serious	Not serious	Not serious	Low	123
12 weeks–6 months	None						
6 months–2 years	1 observational study	None	Not serious	Not serious	Not serious	Low	44
Over 2 years	None						
*BPI*							
0–1 week	1 observational study	None	Not serious	Not serious	Not serious	Low	64
1–12 weeks	None						
12 weeks–6 months	1 observational study	None	Not serious	Not serious	Not serious	Low	862
6 months–2 years	None						
Over 2 years	None						
*HOOS*							
0–1 week	None						
1–12 weeks	None						
12 weeks–6 months	None						
6 months–2 years	1 observational study	None	Not serious	Not serious	Not serious	Low	140
Over 2 years	None						
*Other*							
0–1 week	None						
1–12 weeks	None						
12 weeks–6 months	None						
6 months–2 years	1 observational study	None	Not serious	Serious	Not serious	Very low	441
Over 2 years	None						

*WOMAC* Western Ontario and McMaster University Osteoarthritis Index, *VAS*Visual analogue scale, *SF-36* Short-form health survey, *BPI* Brief pain index, *HOOS* Hip Dysfunction and Osteoarthritis Outcome Score

**Table 5:** GRADE pain and function

Certainty assessment							No. of patients
Outcome measure	Study design	Risk of Bias	Inconsistency	Indirectness	Imprecision	Quality	
*Pain and Function*							
WOMAC							
0–1 week	None						
1–12 weeks	None						
12 weeks–6 months	None						
6 months–2 years	1 observational Study	None	Not serious	Not serious	Not serious	Low	677
Over 2 years	None						

*WOMAC* Western Ontario and McMaster University Osteoarthritis Index

Overall, low GRADE of evidence was found for the main functional measures (WOMAC PF, SF-36 PCS, HHS) and pain measures (WOMAC pain, VAS) showing preoperative anxiety and depression negatively impact postoperative pain and function. Studies were downgraded primarily due to indirectness between psychological variables and desired outcome measures. Singh et al., which assessed both pain and function, was downgraded because a valid scale was not used to evaluate a postoperative outcome [22]. Rather, participants were asked one question regarding their hip pain and function. Negrini et al. [15] sought to determine the influence of depression and anxiety on gait speed; however, participants included did not have preoperative scores which qualified as abnormal, thus the study was downgraded for indirectness. Lastly, Mercurio et al. [14] was downgraded for failing to provide outcome results for intermediate timepoints.

## Discussion

In this systematic review, we investigated the relationship between patient preoperative psychological factors and postoperative THA outcomes. We found preoperative depression and anxiety to be significant predictors of postoperative pain and decreased function. Low self-efficacy was also related to impaired hip function after surgery. All other psychological variables had conflicting results and a smaller sample size in terms of both the number of studies and patients assessed. More trials evaluating the influence of pain catastrophizing, resilience and pessimism could create a clearer picture of the relationship between these psychological variables and THA outcomes. For example, lower resilience in patients with pelvic or extremity fractures has been associated with worse postoperative outcomes and increased opioid consumption [29].

Several earlier reviews have been completed on the topic. A similar 2011 systematic review by Vissers et al. found limited to no evidence that psychological factors predict THA outcomes [30]. However, only nine studies were evaluated with a small overall sample size. Within the last decade, more studies have been conducted linking psychological variables and THA outcomes. In 2018, Bay and colleagues conducted a systematic review evaluating the effectiveness of psychological interventions prior to total hip and knee arthroplasties [31]. While only two of seven randomized clinical trials demonstrated a benefit of presurgical interventions, the results may have been skewed since the studies did not specifically target patients based on preoperative psychological status. Additionally, the studies included in the review lacked sufficient sample sizes.

Although THA may be more complicated for those with comorbidities, the surgery remains important for all populations [32]. Despite positive postoperative outcomes for patients with better preoperative psychological status, most studies reported overall improvements in net pain and function after THA regardless of psychological status [5, 9, 11, 19, 23]. Therefore, THA should continue to be performed on all patients who qualify.

Our systematic review has some limitations. First, all included publications were observational trials and several failed to adjust for confounders such as age, sex and preoperative pain or function. Second, the studies relied on self-report questionnaires or patient reported outcomes for the assessment of psychological status and postoperative state. As a result, potential response bias was difficult to account for. Lastly, we were unable to perform a meta-analysis because of study heterogeneity, specifically differing psychological variables, outcomes measures and varied follow-up times. The study also has several strengths. First, we performed a comprehensive search of large databases and subsequently hand-searched reference lists for additional articles. As a result, we likely included all relevant papers on the study topic. Second, data were thoroughly extracted from all included articles to fully understand the scope of the results and cross-checked for accuracy. Third, the PRISMA guidelines were followed ensuring proper conduct and reporting. Lastly, we performed a risk of bias assessment and GRADE assessment which allowed us to account for method quality and evidence consistency for all included studies.

Moving forward, assessing the preoperative psychological status of patients undergoing THA may help physicians manage expectations of surgical outcomes. A recent study by Geng et al. found depressed patients who underwent psychological therapy had significantly improved postoperative pain and function compared to a control group six months after TKA [33]. Therefore, the evidence suggests preoperative assessment of psychological diagnoses for patients undergoing TJA and treatment for underlying disorders can improve outcomes. Future randomized studies investigating the role of preoperative psychological comorbidities on surgical outcomes after THA would provide additional insight on the topic. Lastly, the literature would benefit from further studies to determine whether routinely used outcome metrics in this patient population are sensitive and specific enough to screen for and monitor changes in specific psychological characteristics.

## Conclusions

We found preoperative psychological variables, mainly depression and anxiety, were predictive of postoperative pain and function following THA. The findings indicate assessing the psychological status of patients prior to surgery can help both patients and physicians better prepare for potential outcomes of THA. Future studies should investigate whether addressing and treating psychological factors prior to surgery improve postoperative outcomes.

## Supplementary Information

**Additional file 1**. PRISMA Checklist

**Additional file 2**. Summary of characteristics of included studies. Abbreviations: WOMAC, Western Ontario and McMaster University Osteoarthritis Index; Pain, pain subscale; PF, physical functioning subscale; SF-36 PCS, Short-Form Health Survey Physical Component Score; HHS, Harris Hip Score; HOOS, Hip Dysfunction and Osteoarthritis Outcome Score; VAS, Visual Analogue Scale; PROMIS-10, Patient Reported Outcomes Measurement Information System-10; BPI, Brief Pain Index; BPI-SF, Brief Pain Index - Short Form; OHS, Oxford Hip Score; HADS, Hospital Anxiety and Depression Scale; CES-D, Center for Epidemiological Studies-Depression; STAI, Spielberger Trait Anxiety Inventory; RS-11, Resilience Scale; BRS, Brief Resilience Scale; FPI-R, Freiburg Personality Inventory - Revised; GSES, General Self-Efficacy Scale; EQ-5D, EuroQol Five-Dimension Index; SF-36 MHS, Short-Form Health Survey Mental Health Score; BSI, Brief Symptom Inventory; BDI, Beck Depression Inventory; PHQ-9, Patient Health Questionnaire; CSQ-RF, Coping Strategies Questionnaire-Revised Form; SFQ, Surgical Fear Questionnaire; LOT-R, Life Orientation Test-Revised; SOMS-2, Screening of Somatoform Disorders; PBQ, Pain Belief Questionnaire; POMS, Profile of Mood States; TCI-R, Temperament and Character Inventory - Revised; MMPI, Minnesota Multiphasic Personality Inventory; SF-36 MCS, Short-Form Health Survey Mental Component Score. * indicates missing or incomplete follow-up data

## Data Availability

All data generated or analyzed during this study are included in this published article.
