# Supplementary material for: Systematic review: preoperative psychological factors and total hip arthroplasty outcomes
Source: J Orthop Surg Res. 2022 Oct 17;17:457. doi: 10.1186/s13018-022-03355-3 (PMC9575292; doi:10.1186/s13018-022-03355-3)
Supplement: Supplementary file 2 — Additional file 2. Summary of characteristics of included studies. Abbreviations: WOMAC, Western Ontario and McMaster University Osteoarthritis Index; Pain, pain subscale; PF, physical functioning subscale; SF-36 PCS, Short-Form Health Survey Physical Component Score; HHS, Harris Hip Score; HOOS, Hip Dysfunction and Osteoarthritis Outcome Score; VAS, Visual Analogue Scale; PROMIS-10, Patient Reported Outcomes Measurement Information System-10; BPI, Brief Pain Index; BPI-SF, Brief Pain Index - Short Form; OHS, Oxford Hip Score; HADS, Hospital Anxiety and Depression Scale; CES-D, Center for Epidemiological Studies-Depression; STAI, Spielberger Trait Anxiety Inventory; RS-11, Resilience Scale; BRS, Brief Resilience Scale; FPI-R, Freiburg Personality Inventory - Revised; GSES, General Self-Efficacy Scale; EQ-5D, EuroQol Five-Dimension Index; SF-36 MHS, Short-Form Health Survey Mental Health Score; BSI, Brief Symptom Inventory; BDI, Beck Depression Inventory; PHQ-9, Patient Health Questionnaire; CSQ-RF, Coping Strategies Questionnaire-Revised Form; SFQ, Surgical Fear Questionnaire; LOT-R, Life Orientation Test-Revised; SOMS-2, Screening of Somatoform Disorders; PBQ, Pain Belief Questionnaire; POMS, Profile of Mood States; TCI-R, Temperament and Character Inventory - Revised; MMPI, Minnesota Multiphasic Personality Inventory; SF-36 MCS, Short-Form Health Survey Mental Component Score. * indicates missing or incomplete follow-up data [file 13018_2022_3355_MOESM2_ESM.docx]

| **First Author, Year** | **Study Design** | **Study Location** | **Follow Up Duration** | **Mean participant age (years)** | **Sex F (%)** | **Psychological Variable, Present:Absent** | **Participants** | **Dropouts After Baseline** | **Outcome** | **Psychological Variable** |
| --- | --- | --- | --- | --- | --- | --- | --- | --- | --- | --- |
| Badura-Brzoza, K. 2008 | Prospective Cohort | Poland | 6 months | 59.0 | 108 (58.7) | NR | 184 | NR | Function (SF-36 PCS) | Depression (HADS)  Anxiety (HADS) |
| Benditz, A. 2017 | Prospective Cohort | Germany | 1,5 weeks | 62.2 | 27 (54.0) | 25:25 | 50 | 11 | Function (HHS) | Depression (CES-D)  Anxiety (STAI) Resilience (RS-11)  Personality (FPI-R) |
| Brembo, E.A. 2017 | Prospective Cohort | Norway | 3 months | 69.3 | 159 (71.3) | NR | 223 | 27 | Function (WOMAC PF) Pain (WOMAC pain) | Self-efficacy (GSES) |
| Duivenvoorden, T. 2013 | Prospective Cohort | Netherlands | 3*,12 months | 67.9 | 89 (63.6) | Anxious 31:109 Depressed 37:103 | 140 | 63 | Function (HOOS) Pain (HOOS) | Depression (HADS)  Anxiety (HADS) |
| Etcheson, J.I. 2018 | Retrospective Cohort | USA | 48 hours | 67.1 | 65 (69.9) | 48:45 | 93 | NR | Pain (VAS) | Depression (Database) |
| Galea, V.P. 2019 | Prospective Cohort | USA | 3 months, 1,3*,5*,7 years | 61.3 | 482 (49.4) | 333:643 | 976 | 349 | Function (SF-36 PCS/ HHS) | Depression (EQ-5D)  Anxiety (EQ-5D) Self Care (EQ-5D) |
| Hassett, A.L. 2018 | Prospective Cohort | USA | 3*,6 months | 71.0 | NR | 72:790 | 862 | NR | Pain (BPI) | Depression (HADS)  Anxiety (HADS) |
| Hossain, M. 2011 | Retrospective Cohort | UK | 1,5 years | 67.0 | NR | 203:705 | 908 | 293 | Function (OHS) | Distress (SF-36 MHS) |
| Jaiswal, P. 2019 | Prospective Cohort | Canada | 1 year | 67.5 | 394 (58.2) | NR | 677 | 99 | Function and Pain (WOMAC) | Mental Health (SF-36 MHS) |
| Lindner, M. 2018 | Prospective Cohort | Germany | 6,12 weeks | 67.4 | 23 (52.3) | NR | 44 | NR | Function (WOMAC PF) Pain (WOMAC pain) | Depression (BSI)  Anxiety (BSI) |
| Mercurio, M. 2020 | Prospective Cohort | Italy | 5 days*, 1*,3*,6*,12 months | 65.6 | 18 (45.0) | NR | 40 | NR | Function (WOMAC PF/HHS/SF-12 PCS) Pain (WOMAC pain/VAS) | Depression (HADS)  Anxiety (HADS) Personality (TCI-R) |
| Negrini, F. 2020 | Prospective Cohort | Italy | 3,12 days | 62.8 | 22 (55.0) | NR | 40 | NR | Function (Gait Speed) | Depression (BDI)  Anxiety (STAI) |
| Pinto, P.R. 2017 | Prospective Cohort | Portugal | 48 hours | 63.3 | 38 (59.4) | NR | 64 | NR | Pain (BPI-SF) | Depression (HADS)  Anxiety (HADS) Pain Catastrophizing (CSQ-RF)  Surgical Fear (SFQ) Optimism (LOT-R) |
| Quintana, J.M. 2009 | Prospective Cohort | Spain | 6 months, 2 years | 69.3 | 287 (36.4) | NR | 788 | 198 | Function (WOMAC PF) Pain (WOMAC pain) | Mental Health (SF-36 MHS) |
| Rasouli, M.R. 2016 | Retrospective Cohort | USA | N/A | 62.1 | 463 (48.0) | 62:902 | 964 | NR | Complications | Depression (Database) Anxiety (Database) |
| Riediger, W. 2010 | Prospective Cohort | Germany | 2 months | 68.0 | NR | Depressed 15:64  Somatoform 19:60 | 79 | 22 | Function (WOMAC PF/SF-36 PCS) Pain (WOMAC pain/SF-36 pain) | Depression (HADS)  Somatization (SOMS-2) Pain Beliefs (PBQ) |
| Rolfson, O. 2009 | Retrospective Cohort | Sweden | 1 year | 69.0 | 3506 (56.9) | 2628:3530 | 6158 | NR | Pain (VAS) | Depression (EQ-5D) Anxiety (EQ-5D) |
| Salmon, P. 2001 | Prospective Cohort | UK | 1,6 months | 69.0 | 63 (61.8) | NR | 102 | 8 | Function (WOMAC PF) | Depression (POMS) Anxiety (POMS) |
| Singh, J.A. 2016 | Retrospective Cohort | USA | 2 years | 67.4 | 252 (57.1) | 113:328 | 441 | 47 | Function ("How would you rate your hip function?") Pain ("How much pain do you have in your hip?") | Pessimism (MMPI) |
| Tarakji, B.A. 2018 | Retrospective Cohort | USA | 3,12 months | NR | NR | 12:32 | 44 | NR | Function (SF-36 PCS) Pain (SF-36 pain) | Depression (SF-36 MCS) |
| Trinh, J.Q. 2020 | Prospective Cohort | USA | 1 year | NR | NR | NR | 48 | NR | Function (PROMIS-10) | Depression (PHQ-9) Resilience (BRS) |

**Additional File 1/Table 1:** Summary of characteristics of included studies.

Abbreviations: WOMAC, Western Ontario and McMaster University Osteoarthritis Index; Pain, pain subscale; PF, physical functioning subscale; SF-36 PCS, Short-Form Health Survey Physical Component Score; HHS, Harris Hip Score; HOOS, Hip Dysfunction and Osteoarthritis Outcome Score; VAS, Visual Analogue Scale; PROMIS-10, Patient Reported Outcomes Measurement Information System-10; BPI, Brief Pain Index; BPI-SF, Brief Pain Index - Short Form; OHS, Oxford Hip Score; HADS, Hospital Anxiety and Depression Scale; CES-D, Center for Epidemiological Studies-Depression; STAI, Spielberger Trait Anxiety Inventory; RS-11, Resilience Scale; BRS, Brief Resilience Scale; FPI-R, Freiburg Personality Inventory - Revised; GSES, General Self-Efficacy Scale; EQ-5D, EuroQol Five-Dimension Index; SF-36 MHS, Short-Form Health Survey Mental Health Score; BSI, Brief Symptom Inventory; BDI, Beck Depression Inventory; PHQ-9, Patient Health Questionnaire; CSQ-RF, Coping Strategies Questionnaire-Revised Form; SFQ, Surgical Fear Questionnaire; LOT-R, Life Orientation Test-Revised; SOMS-2, Screening of Somatoform Disorders; PBQ, Pain Belief Questionnaire; POMS, Profile of Mood States; TCI-R, Temperament and Character Inventory - Revised; MMPI, Minnesota Multiphasic Personality Inventory; SF-36 MCS, Short-Form Health Survey Mental Component Score.

* indicates missing or incomplete follow-up data
